# Learning to combine top-down context and feed-forward representations under ambiguity with apical and basal dendrites

**DOI:** 10.1093/cercor/bhaf134

**Published:** 2025-06-23

**Authors:** Nizar Islah, Guillaume Etter, Mashbayar Tugsbayar, Busra Tugce Gurbuz, Blake Richards, Eilif B Muller

**Affiliations:** Centre de Recherche Azrieli du CHU Ste-Justine, Université de Montréal, 3175 Chem. de la Côte-Sainte-Catherine, Montréal H3T 1C5, Quebec, Canada; Département d’informatique et de recherche opérationnelle, Université de Montréal, Pavillon André-Aisenstadt, 2920, chemin de la Tour local 2194, Montréal, H3T 1N8, Québec, Canada; Mila - Quebec AI Institute, 6666 Rue Saint-Urbain, Montréal H2S 3H1, Quebec, Canada; Centre de Recherche Azrieli du CHU Ste-Justine, Université de Montréal, 3175 Chem. de la Côte-Sainte-Catherine, Montréal H3T 1C5, Quebec, Canada; Mila - Quebec AI Institute, 6666 Rue Saint-Urbain, Montréal H2S 3H1, Quebec, Canada; Mila - Quebec AI Institute, 6666 Rue Saint-Urbain, Montréal H2S 3H1, Quebec, Canada; Department of Neurology and Neurosurgery, McGill University, 1033 Pine Avenue West, Montréal H3A 1A1, Quebec, Canada; Montreal Neurological Institute, McGill University, 3801 Rue University, Montréal H3A 2B4, Quebec, Canada; Centre de Recherche Azrieli du CHU Ste-Justine, Université de Montréal, 3175 Chem. de la Côte-Sainte-Catherine, Montréal H3T 1C5, Quebec, Canada; Mila - Quebec AI Institute, 6666 Rue Saint-Urbain, Montréal H2S 3H1, Quebec, Canada; Montreal Neurological Institute, McGill University, 3801 Rue University, Montréal H3A 2B4, Quebec, Canada; Department of Quantitative Life Sciences, McGill University, 550 Sherbrooke W., Montréal H3A 1E3, Quebec, Canada; Mila - Quebec AI Institute, 6666 Rue Saint-Urbain, Montréal H2S 3H1, Quebec, Canada; Department of Neurology and Neurosurgery, McGill University, 1033 Pine Avenue West, Montréal H3A 1A1, Quebec, Canada; Montreal Neurological Institute, McGill University, 3801 Rue University, Montréal H3A 2B4, Quebec, Canada; School of Computer Science, McGill University, 3480 Rue University, Montréal H3A 2A7, Quebec, Canada; CIFAR Learning in Machines and Brains Program, 661 University Ave., Suite 505, Toronto M5G 1M1, Ontario, Canada; Centre de Recherche Azrieli du CHU Ste-Justine, Université de Montréal, 3175 Chem. de la Côte-Sainte-Catherine, Montréal H3T 1C5, Quebec, Canada; Département d’informatique et de recherche opérationnelle, Université de Montréal, Pavillon André-Aisenstadt, 2920, chemin de la Tour local 2194, Montréal, H3T 1N8, Québec, Canada; Mila - Quebec AI Institute, 6666 Rue Saint-Urbain, Montréal H2S 3H1, Quebec, Canada; Département de neurosciences, Faculté de médecine, Université de Montréal, Pavillon Paul-G.-Desmarais, 2960 chemin de la Tour local 111, Montréal H3T 1J4, Québec, Canada

**Keywords:** apical dendrite, contextual integration, pyramidal neuron, top-down

## Abstract

One of the hallmark features of neocortical anatomy is the presence of extensive top-down projections into primary sensory areas. It is hypothesized that one of the roles of these top-down projections is to carry contextual information that helps animals to resolve ambiguities in sensory data. One proposed mechanism of contextual integration is a combination of input streams at distinct apical and basal dendrites of pyramidal neurons. Computationally, however, it is yet to be demonstrated how such an architecture could leverage distinct compartments for flexible contextual integration and sensory processing. Here, we implement a deep neural network with distinct apical and basal compartments that integrates (a) contextual information from top-down projections to apical compartments and (b) sensory representations driven by bottom-up projections to basal compartments. In addition, we develop a new contextual integration task using generative modeling. The performance of deep neural networks augmented with our “apical prior” exceeds that of single-compartment networks. We find that a sparse subset of neurons of the context-relevant categories receive the largest top-down signals. We further show that this sparse gain modulation is necessary. Altogether, this suggests that the “apical prior” could be key for handling the ambiguities that animals encounter in the real world.

## Introduction

Accurate perception relies on appropriate integration of context, as sensory signals contain incomplete or ambiguous information (Mumford 1992; Rao and Ballard 1999). Contextual information can be derived from a number of sources, including the spatial and temporal domains (Eichenbaum 2017), task demands (Gilbert and Li 2013), as well as other sensory modalities.

Several key neuroanatomical and cellular features could support the computations associated with context integration. In the mammalian brain, context has been proposed to be conveyed by top-down feedback pathways, which are abundant in sensory regions of the neocortex (Felleman and Van Essen 1991; Markov 2014; Harris 2019). Perturbations of top-down pathways from higher order areas have been associated with delayed object recognition (Kar and DiCarlo 2021) as well as disrupted stimulus response curves in lower order regions (Wang et al. 2007; Nassi et al. 2013), which may reflect deficits in contextual processing. Several feed-forward computational models of perception have implemented top-down pathways to enable object recognition of occluded images (George 2017; Spoerer et al. 2017) as well as context-invariant perception (Naumann et al. 2022), and context-dependent gating for multitask learning (Masse et al. 2018). Interestingly, an ANN model augmented with hierarchical top-down neuron masking (a less expressive form of top-down modulation) also improved out-of-distribution robustness (Liu 2023).

In the neocortex, pyramidal neurons are thought to integrate feedforward and top-down, feedback information at their basal and apical dendrites, respectively (Larkum et al. 2007; Spruston 2008). Notably, feedback has been proposed to modulate rather than drive neuronal activities (Sherman and Guillery 1998). This idea is supported by the unique morphological and physiological properties of pyramidal neurons, as activity in apical dendrites in vivo is generally not sufficient to drive somatic output responses alone (Stuart and Spruston 1998; Larkum et al. 1999; Larkum 2013; Jarvis et al. 2018). However, activation of the apical compartment has been shown to act as a gain modulator, amplifying concurrent basal activity (Larkum et al. 2004), serving as a potential mechanism for contextual information. While the physiological properties of pyramidal neurons have been described extensively (see Spruston (2008) for review), there is no consensus on the exact mechanism by which top-down signals from higher order regions modulate somatic firing rates and update sensory representations.

Here, we propose a deep neural network architecture in which neurons are augmented specifically with apical and basal compartments and whose computation respects known biophysical properties of pyramidal neurons. We design a training paradigm such that the model updates its representations of sensory inputs arriving at the basal compartment according to the top-down contextual information arriving at the apical compartment.

In tandem with the proposed framework, we develop a multiscenario task with a dataset containing ambiguous images that are mixtures of two image categories, in which ambiguity can only be correctly resolved by integrating contextual information with sensory representations.

We consider two versions of the problem setting. The first simplifies the contextual representation, which is given directly via a top-down oracle, and the model only needs to learn the apical parameters to correctly update the somatic activity. In the second, more challenging one, the model must use the temporal sequence as context and learn the appropriate contextual representations, along with the apical parameters to update somatic activity and ultimately solve the task.

We show that the model learns to use apical modulation, driven by contextual inputs, to resolve sensory ambiguities. To gain insights into the learned mechanisms of top-down modulation, we applied a standard method from the explainable artificial intelligence (AI) literature to identify the relative contributions of individual neurons and their apical compartments to network function. We identified a subset of neurons that are highly relevant for the contextually relevant category, and selectively amplified by top-down signals on apical dendrites.

Our results show that apical gain modulation of somatic activity by contextual information is a simple yet efficient way to solve new tasks, such as resolving perceptual ambiguity. Moreover, the approach is flexible, because it is achieved without altering what the model has previously learned, as it does not introduce or modify any synapses in the feed-forward pathway.

These findings provide a candidate mechanism for how neocortical pyramidal neurons integrate top-down contextual information at their apical dendrites to support robust perception in the face of ambiguous data.

## Materials and methods

### Ambiguous dataset generation

We developed a new visual contextual integration task composed of multiple scenarios that incorporate varying levels of ambiguity of sensory input and informativeness of contextual information.

To create the image dataset for our task, we used a digit-conditioned generative approach based on conditional variational autoencoders (CVAEs). First, we train a CVAE on standard a image dataset, either MNIST for digits or EMNIST for characters (Table 1) (Sohn et al. 2015; Doersch 2021). We then use the learned latent space along with a digit-conditioned input vector to generate images that are ambiguous between two digits by interpolating between the one-hot vectors of the two digits.

**Table 1:** Ambiguous MNIST and EMNIST (aMNIST, aEMNIST) test set classification accuracy across input-context scenarios with the Hadamard integration rule. Results are expressed for each scenario as mean % $\pm $ standard deviation with 3 random seeds.

Task	Context signal	ambig (Baseline)	ambig.Match	unambig.Match	unambig.Irrel	ambig.Irrel	unambig.Contra
aMNIST	oracle (one-hot)	$46.3 \pm 0.3$	$98.5 \pm 0.3$	$98.5 \pm 0.1$	$97.9 \pm 0.1$	$46.0 \pm 0.8$	$96.0 \pm 0.4$
aEMNIST	oracle (one-hot)	$46.5 \pm 0.5$	$94.2 \pm 1.4$	$98.9 \pm 0.2$	$96.2 \pm 0.4$	$47.1 \pm 0.8$	$94.9 \pm 0.5$

We then assess the ambiguous samples using a trained classifier on the original dataset (either MNIST or EMNIST), and discard images that are outside the $50\% (\pm 5\%)$ decision boundary between the two digits. We organize our dataset of images into triplets, where two images are unambiguous from digits $y_{0}, y_{1}$, and the third is generated by the CVAE to be ambiguous between $y_{0}, y_{1}$. This dataset format simplifies the implementation of our task setup.

### Contextual integration model architecture

The contextual integration model consists of three components: the pretrained backbone that computes representations of input images, the readout network for classifying their respective representations, and the top-down network that modulates the intermediate representations from the pretrained backbone based on contextual information. The backbone is implemented as a convolutional variational autoencoder (VAE), and was pretrained on unambiguous characters with the standard ELBO loss. VAEs are known to learn a smooth latent space, which we assume here to be an important ingredient to allow learning of top-down contextual modulations. Next, we freeze the VAE weights and train a readout (multilayer perceptron [MLP]) on the VAEs latent representations of images with a classification objective (cross entropy loss). Finally, we freeze these components and train the top-down network in two contextual tasks, with oracle and temporal context, respectively, as described in the next sections. The top-down network $g(\cdot )$ is implemented as an MLP mapping the concatenated VAE latents and context vector to apical activations (Equation 8), which then modulate the VAEs intermediate representation. Various modulation functions (Hadamard, additive, concatenation) are explored in the main text.

#### Oracle context

In the oracle case, context signals are provided to the top-down network $g$ in the form of a one-hot vector:


(1) \begin{align*}& \mathbb{1}_{y}(c_{i}):=\begin{cases} 1, & \text{if}\ {i = y}.\\ 0, & \textrm{otherwise} \end{cases}\end{align*}

$\forall i \in{1\dots C}$ , where C is the number of classes, $|\mathbf{c}| = C$.

#### Temporal context

In the temporal case, the information is presented in a sequence, and preceding elements determine the contextual information available to the model at the current time $t$. Therefore, we leverage a Gated Recurrent Unit (GRU) network (Cho 2014) to dynamically capture and represent the temporal dependencies in the sequence. We trained our GRU to predict the modulo 10 sum of MNIST digits in a sequence of two digits, reflecting the task in our sequential MNIST/EMNIST dataset. An important detail is that the input to the GRU at time $t$ is not the image $x_{t}$, but rather the latent $\mathbf{Ub}_{t}$, encoded by the pretrained backbone. After processing a sequence of digits ($x_{t-2}, x_{t-1}$), the output state $o_{t}$ of the GRU was then used as a top-down context signal, in place of the one-hot context from the oracle case. We used a hidden state of size 128 for the GRU.

### Training

#### Loss function

The top-down network $g_{\Theta _{a}}$ is explicitly trained to minimize a loss function jointly across the following five conditions: ambiguous input with matching context (ambig.Match), unambiguous input with matching context (unambig.Match), unambiguous input with irrelevant context (unambig.Irrel), ambiguous input with irrelevant context (ambig.Irrel), and unambiguous input with contradictory context (unambig.Contra). We use mean squared error as the loss criterion between the predicted (computed as in equation 7) and the target latent representations. Each minibatch is sampled from dataset $D$, and training data for each condition are equally balanced in the minibatch.


(2) \begin{align*}& \mathbf{L}(\Theta_{a};D) = \mathbb{E}_{x\sim D, c_{match},c_{irrel}\sim C} \left[ \sum_{s=1}^{5} ||\mu_{s}^{*}-\hat{\mu_{s}}||_{2}^{2} \right],\end{align*}

where $x = ((x_{0},y_{0}),x_{ambig},(x_{1},y_{1}))$, and $x_{0},x_{1}$ are unambiguous images sampled from $D$. The optimum of $\Theta _{a}$ is given by


(3) \begin{align*}& \Theta^{*}_{a} = argmin_{\Theta_{a}} L(\Theta_{a};D).\end{align*}

Here, $\mu _{s}^{*}$ is the target representation, which is for each scenario and context:


(4) \begin{align*}& \mu_{s}^{*}:=\begin{cases} \mathbf{U}\mathbf{\sigma(b_{c_{match}})}, & \textrm{if}\ s\in \{1\} \\ \mathbf{U}\mathbf{\sigma(b_{unambig})}, & \textrm{if}\ s\in \{2,3\} \\ \mathbf{U}\mathbf{\sigma(b_{ambig})}, & \textrm{if}\ s \in \{4\} \\ \mathbf{U}\mathbf{\sigma(b_{c_{match}})}, & \textrm{if}\ s\in \{5\} \end{cases},\end{align*}

where $b_{c} = f(x_{c}|\Theta _{b})$ and $b_{unambig} = f([x_{0},x_{1}]|\Theta _{b})$. Predicted latents are given by


(5) \begin{align*}& \hat{\mu}_{s}:=\begin{cases} \mathbf{U}\mathbf{\sigma(b_{ambig})\odot(1+\sigma(a_{b_{ambig},c_{match}}))}, & \textrm{if}\ s\in \{1\} \\ \mathbf{U}\mathbf{\sigma(b_{unambig})\odot(1+\sigma(a_{b_{unambig},c_{match}}})), & \textrm{if}\ s\in \{2\} \\ \mathbf{U}\mathbf{\sigma(b_{unambig})\odot(1+\sigma(a_{b_{unambig},c_{irrel}}})), & \textrm{if}\ s\in \{3\} \\ \mathbf{U}\mathbf{\sigma(b_{ambig})\odot(1+\sigma(a_{b_{ambig},c_{irrel}}))}, & \textrm{if}\ s \in \{4\} \\ \mathbf{U}\mathbf{\sigma(b_{unambig})\odot(1+\sigma(a_{b_{unambig},c_{contra}}})), & \textrm{if}\ s\in \{5\} \\ \end{cases},\end{align*}

where $\mathbf{a_{b,c}} = {g(\mathbf{U \sigma (b)\oplus c}|\Theta _{a})}$.




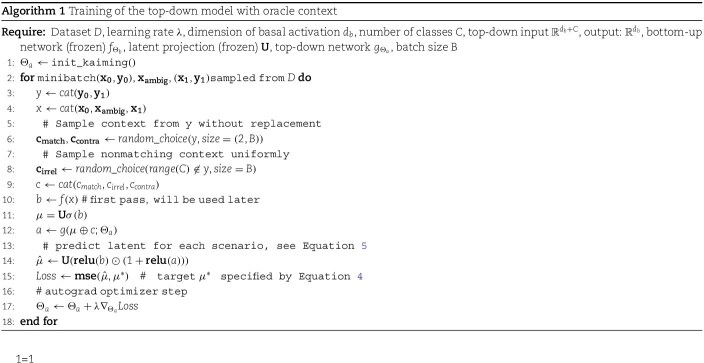



#### Layer-wise relevance propagation analysis

**Fig. 1:**
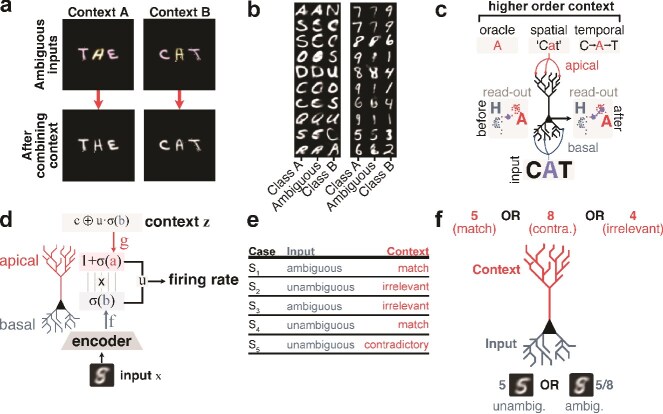
Functional model of context integration in apical dendrites. (a), in our experimental paradigm, ambiguous characters (in yellow, top panels) are resolved by combining context signals (bottom panels). (b), examples of unambiguous pairs and resulting ambiguous characters generated using EMNIST (left) and MNIST (right). (c), model of pyramidal neurons with distinct dendritic compartments trained to solve a multiscenario contextual integration task with ambiguity. Before top-down modulation, 2 predictions have equal probability. After top-down modulation, context contributes to favoring one unambiguous representation. (d), implementation of dendritic integration in artificial neurons with apical and basal compartments. $f$ represents the feedforward mapping onto the basal compartment parameterized by the pretrained backbone, and $g$ the contextual top-down mapping onto the apical compartment, parameterized by its own neural network. The combination of the outputs $b$ and $a$ of $f$ and $g$, respectively, is applied neuron-wise via the multiplicative interaction $\sigma (b) * (1 + \sigma (a))$. (e), distinct combinations of input and context used to train the top-down network $g$. (f), possible combinations of input and context used during training and analysis.

We applied layer-wise relevance propagation (LRP) (Bach 2015) to identify specific neurons whose activity, as described by $\mathbf{h}$ in our model, contributed most to the category readouts. For this analysis, we trained a readout to classify the standard categories (ie digits 0 to 9) as well as all possible ambiguous pairs (ie 3/5, 5/8, 1/7, etc.), which for MNIST gives a total number of categories $C=55$. To identify the subset of neurons that are most relevant for a given class $i$, we sorted neurons by highest to lowest average relevance per class. We then computed the normalized cumulative sum of relevance of neurons, and define $S_{i}$ as the minimum set of neurons required to obtained 95% of the total LRP for each class $i$, where $i \in{0,1, \cdots , C}$ (Fig. 1d). We computed the separability between sets $S_{i}$, $S_{j}$ as $1-|S_{i} \cap S_{j}| / |S_{i} \cup S_{j}|$ (Fig. 1a).

### Statistical analysis

Parametric tests where used when data distribution was normal and variance homogeneous, otherwise nonparametric tests were used and reported when appropriate. **Meaning of abbreviated statistical terms**. 1-ANOVA, one-way ANOVA; 2-ANOVA, two-way ANOVA. Error bars and bands represent standard error of the mean, unless stated otherwise. ^*^, $P$ < 0.05; ^*^^*^, $P$ < 0.01; ^*^^*^^*^, $P$ < 0.001; ^*^^*^^*^^*^, $P$ < 0.0001; n.s., not significant.

## Results

### Functional model of context integration in apical dendrites

To model how apical dendrites integrate context to refine perceptual representations, we first developed a multiscenario contextual integration task where correct classification of ambiguous characters requires appropriate integration of contextual cues (Fig. 1a). To create the task, we trained a generative model on handwritten digits and letters (MNIST and EMNIST, respectively) to construct a dataset with highly ambiguous characters. We used the generative model to synthesize images that are a mixture of two classes (see Methods). The ambiguity of the synthesized images was empirically quantified via softmax on classifier predictions, where we select images near 50% for two classes (Fig. 1b). Inspired by anatomical features of pyramidal neurons, we next developed a model that implements distinct dendritic compartments, a basal compartment for integrating sensory information and an apical compartment for integrating contextual information (Fig. 1c). In the model, a population of $n$ pyramidal neurons has two sources of inputs, represented as vectors: a set of basal inputs, $\mathbf{b} = [b_{1}, \ldots , b_{n}]$, representing the sensory stream, and a set of apical inputs, $\mathbf{a} = [a_{1}, \ldots , a_{n}]$, representing the top-down stream. Input images, $\mathbf{x}$, are encoded by a pretrained network $f$, with parameters $\Theta _{b}$, where $f$ is a convolutional neural network reflecting initial stages of sensory processing (see Methods for more details on how the encoder is pretrained). The output of $f(x)$ is the basal vector $\mathbf{b}$.

The firing rate of pyramidal neurons, $\mathbf{h} = [h_{1}, \ldots , h_{n}]$, is then determined by a thresholded neuron-wise (element-wise) combination of the basal and apical input vectors:


(6) \begin{align*}& \mathbf{h} = \sigma(\mathbf{b}) \odot (\sigma(\mathbf{a})+1),\end{align*}

where $\odot $ is an neuron-wise multiplication (Hadamard) and $\sigma $ is the rectified linear unit (ReLU) activation function implementing basal and apical nonlinearities, respectively. As can be seen from equation 6, the impact of the apical input vector, $\mathbf{a}$, on the firing rate, $\mathbf{h}$, is determined by $\sigma (\mathbf{a})+1$. As such, the apical activity serves as a thresholded neuron-wise gain modulator of the basal activity, in-line with previous experimental reports of a multiplicative role for apical inputs (Waters et al. 2003), an architectural bias of neocortical networks we refer to here as the “apical prior.” Under these constraints, apical inputs affect neuronal activity only when they exceed their activation threshold (when $a> 0$), analogous to observations that apical inputs influence somatic spiking only if they evoke a dendritic calcium spike (Larkum et al. 1999). Thus, this neuron-wise gain modulation of basal representations by the apical compartment via the Hadamard product is an implementation of the “apical prior.”

To monitor the ability of the model to solve the perceptual task, we read-out model predictions from a linear transformation of the pyramidal neurons firing rates:


(7) \begin{align*}& \mathbf{\mu} = \mathbf{U} \mathbf{h},\end{align*}

where $\mu $ represents a higher neuronal population and $\mathbf{U}$ is a linear transformation (to the latent space of the encoder $f$). To obtain the apical inputs $\mathbf{a}$, the context signal, $\mathbf{c}$, is concatenated with $\mu $, and mapped onto the apical compartment using an MLP, $g(\cdot )$, as follows:


(8) \begin{align*}& \mathbf{a} = g(\mathbf{c} \oplus \mathbf{U\sigma{(b)}}),\end{align*}

where $\oplus $ is the concatenation operation, $\sigma $ is the ReLU activation, and $g$ is a MLP with one hidden layer, and its parameters are denoted by $\Theta _{a}$.

As a population, these pyramidal neurons integrate sensory inputs (representations of characters) onto their basal dendritic compartment on one hand, and contextual information onto their apical dendritic compartment on the other hand.

For ambiguous characters, which are a mixture of two categories, a readout of the representations of the model will yield two plausible interpretations, and contextual information is required to resolve the ambiguity. We consider the category that is compatible with the contextual input to be the target (match) category. Conversely, a category that is a plausible interpretation of the input but not compatible with the contextual information is referred to as the contradictory category. We refer to all other categories as irrelevant categories. In total, we consider five possible scenarios. The labels and corresponding descriptions we use for each scenario are provided explicitly below:

- **ambig.Match**: ambiguous input, helpful (matching) context
- **unambig.Irrel**: unambiguous input, irrelevant context
- **ambig.Irrel**: ambiguous input, irrelevant context
- **unambig.Match**: unambiguous input, helpful (matching) context
- **unambig.Contra**: unambiguous input, contradictory context

To test whether the contextual integration model incorporating the “apical prior” can learn apical modulations that are helpful for resolving ambiguous stimuli, we trained the synaptic weights $\Theta _{a}$ with the objective of modulating basal representations to match the target $\mu $ (representation after linear transform). We optimize this objective simultaneously for the set of all input-context scenarios listed above (Fig. 1e and Equation 2) using a gradient descent optimization algorithm (Adam, see pseudocode Algorithm 1).

This setup ensures that the top-down model is trained on both unambiguous and ambiguous examples, and that the model must learn to provide the appropriate modulatory signal only when contextual inputs provide useful information for solving the task, and ignore them when they are irrelevant or contradictory to sensory information. Our proposed implementation reflects the reality that, in the mammalian brain, top-down modulation is available from multiple sources regardless of the ambiguity of the sensory inputs or the relevance of the contextual information.

This suggests an approach where the problem of contextual integration is broken into two parts: (1) how apical dendrites locally learn to use contextual representations $c$ to solve the multiscenario task and (2) how useful contextual representations $c$ can be learned from available sources (eg temporal information).

For the first part, our initial experiments assume that context, $c$, is given in the form of a one-hot vector, a scenario we refer to as the “oracle context” (see Methods). Subsequent experiments for the second part address the problem of learning a representation of context, $c$, taking inspiration from the neocortex, where context is, in part, represented by higher regions with broader windows of temporal integration (Eichenbaum 2017) (Fig. 1c; see Methods).

### Resolving ambiguity in the oracle context

**Fig. 2:**
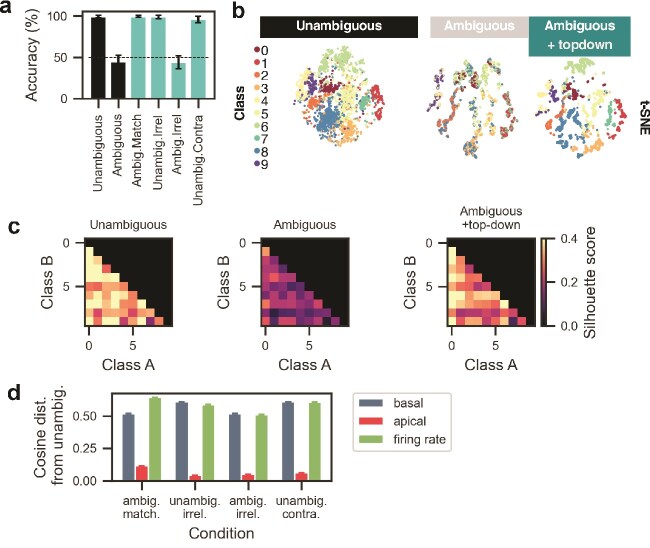
Our task is best solved when top-down signals are integrated correctly. (a), readout test set accuracy without (black) and with (cyan) top-down signals for all input-context combinations. The dashed line corresponds to chance level. (b), low-dimensional projection (t-SNE) of the latent representation, $\mu $, for unambiguous inputs (top), ambiguous inputs before (left), and after (right) top-down modulation. (c), silhouette scores for latent representation, $\mu $, for each class pair and for unambiguous inputs (left), ambiguous inputs before (center), and after (right) top-down modulation. (d), cosine distance between unambiguous activation, $h$, and basal, apical compartments and firing rate in each scenario.

To first test if our model can solve our multiscenario task under contextual input represented as a one-hot vector (oracle context), we trained $g$ on the ambiguous MNIST/EMNIST task with a loss function that includes a loss term for each scenario We assessed performance of the model in each scenario as the accuracy of the readout on the test set, and found significant impact of top-down modulation (1-ANOVA; $F_{5, 1872}$ = 10076.598; $P$ < 0.0001). Specifically, test set accuracy on ambiguous digits was $46.3 \pm 0.3\%$ without top-down modulation, and became significantly higher with top-down modulation ($98.5 \pm 0.3\%$; Tukey test; $P$ < 0.0001) if context was matching, but did not significantly alter performance if context was irrelevant ($43.920 \pm 8.160$; Tukey test; $P$ = 0.4651; $n = 313$ independent replicates; Fig. 2a; Table 2). Importantly, the model did not learn to rely solely on top-down context, since performance remained high when the model was given contradictory context (Fig. 2a; Table 2). To visualize the representations of the model on a population level, we projected un-modulated and modulated outputs $\mathbf{\mu }$ onto a two-dimensional manifold using t-SNE (Maaten (2008); Fig. 2b). Ambiguous images whose representations exhibited high overlap between class-pairs were effectively disentangled after integrating matching contextual inputs (paired t-test, t$_{99}$ = 6.0293, $P$ < 0.0001 for silhouette scores between ambiguous images before and after integrating top-down inputs; Fig. 2c). These results show that our model learned to use contextual inputs provided to apical dendrites to modulate basal activity and resolve ambiguity.

**Table 2:** Sequential aMNIST (seq-aMNIST) test set classification accuracy across input-context scenarios with the Hadamard integration rule. Results are expressed for each scenario as mean % $\pm $ standard deviation. In the temporal case, context signals are provided to the contextual integration network as GRU hidden state representations, conditioned on previous inputs in the sequence. Results are expressed for each scenario as mean % $\pm $ standard deviation with three random seeds.

Task	Context signal	ambig.Match	unambig.Match	unambig.Irrel	ambig.Irrel	unambig.Contra
seq-aMNIST	temporal	96.3 $\pm $ 0.1	99.9 $\pm $ 0.0	99.1 $\pm $ 0.1	48.7 $\pm $ 0.5	99.0 $\pm $ 0.1

Next, we evaluated the importance of having top-down apical compartments to handle contextual inputs compared with only having basal compartments (akin to point-wise spiking neurons; Appendix 1 Table 4). We found that, while there was not a large difference in the other four scenarios, there was an average increase in the performance of 1.9% (MNIST) in the unambig.Contra scenario for the models that had apical compartments compared with those that only had basal compartments.

**Fig. 3:**
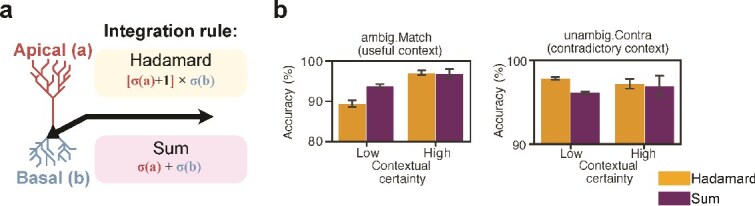
Role of integration rules on ambiguity resolution. (a), comparison between Hadamard and additive integration rules. (b), test set accuracy relative to contextual certainty using either integration rule in conditions where inputs are ambiguous and context is relevant (ambig.Match) or where inputs are unambiguous and context is contradictory to sensory input (unambig.Contra).

Next, focusing our analysis on MNIST, we evaluated the importance of the integration rule that combines basal and apical activities (Fig. 3a, Appendix 1 Table. 4).

We found a significant interaction between the integration rule and contextual certainty (2-ANOVA, F$_{1, 16}$ = 5.4741, $P$ = 0.0346). When inputs are ambiguous and the context is highly certain (context vector with values >0.99 for the target class), we find that the additive and multiplicative (Hadamard) integration rules perform similarly (pairwise t-test, t$_{4}$ = 0.1708, $P$ = 0.8678). For lower levels of certainty (context vector with values <= 0.6 for the target class), we note the following observations: (1) when context is matching, Hadamard integration is outperformed by Sum integration (pairwise t-test, t$_{4}$ = −4.7677, $P$ = 0.0089, and (2) when context is contradictory, Hadamard integration outperforms Sum integration (pairwise t-test, t$_{4}$ = 8.9341, $P$ = 0.0009; Fig. 3b).

### Single neuron mechanisms of top-down modulation

We next sought to understand the computational role of top-down modulation of pyramidal apical dendrites in solving this contextual integration task. To this end, we used a method from the explainable AI literature to rank and identify the neurons most relevant for solving the task. We then compared their activations across the various context-input scenarios. First, to define the most relevant neurons, we employed LRP (Bach 2015); Appendix Fig. 1a), a method in the explainable AI literature to reveal, at each layer, the input’s contribution to the model output. Each neuron receives a score for its contribution to the model output for each image (Appendix Fig. 1b). We then defined the subset of neurons most relevant for predicting a given class, eg class A or class B, as the minimum set of neurons required to account for 95% of total neuronal relevance when processing images from that class (Appendix Fig. 1c,d, see Methods). Similarly, we define subsets of neurons most relevant for predicting that an image is ambiguous between two classes A and B (ambiguous AB). Sets of relevant neurons defined in this way exhibit low overlap between classes (Appendix Fig. 1b to d, see Methods). We observed similar representational separability between classes for other scenarios (Appendix Fig. 1e). Additionally, these sets of relevant neurons are typically sparse and represent $\sim $15% of the total neuronal population (Appendix Fig. 1b,c).

**Fig. 4:**
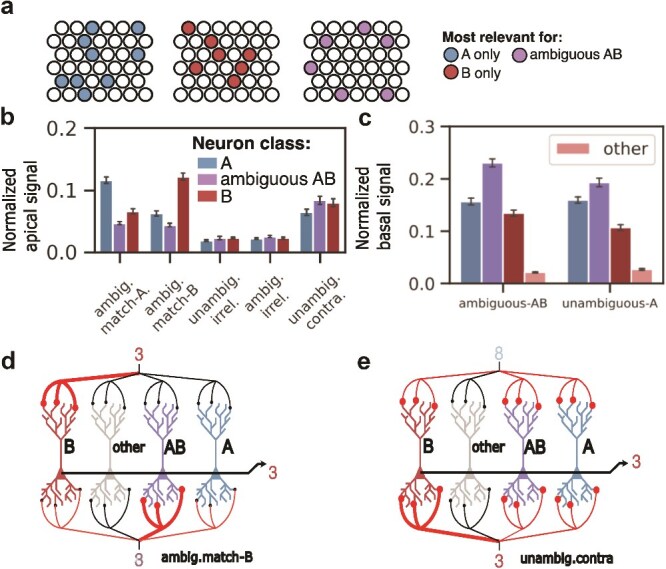
Top-down has information about ambiguity, and, in cases where inputs are ambiguous and context is aligned, resolves ambiguity by amplifying context-relevant neurons. Although in the figure we show multiple apical/basal branches, the apical and basal trees are treated as single units per neuron. (a), using LRP, we identify neurons most relevant for either of two classes (red and blue), or for ambiguous inputs between the two classes (magenta). (b), amplitude of the apical compartment (normalized over all neurons) for each input/context condition. Three distinct classes of neurons are compared: neurons most relevant for unambiguous class A (blue), B (red), or neurons most relevant for the ambiguous AB images (magenta). (c), Normalized basal signal. Left: in case of ambiguous stimuli, both plausible class-subpopulations are active along with the ambiguity detector subpopulation. Right: in case of unambiguous stimuli, we observe a similar distribution, with the context-relevant neurons (class A) having higher average firing rate. (d), a cartoon summary of the observed mechanism for the ambig.match-B case (ambiguous input, contextual input favoring class B), where class B neurons code for “3” (dark red) and class A neurons code for “8” (blue). Context-relevant neurons (dark red) receive the highest amplitude of top-down signals when processing ambiguous input. At basal dendrites, these sub-populations have low but nonzero firing rates, which allows them to be selectively amplified with top-down signals via the Hadamard integration rule, and thus dictate the output representation. (e), for unambiguous input and contradictory context (unambig.contra), top-down input becomes nonspecific and neurons are weakly amplified by the Hadamard rule. The output is then dictated by the neurons receiving the strongest basal input, in this case class B neurons (dark red). Output representations for unambiguous inputs are thus preserved when contextual inputs are added.

It is noteworthy that our model is not explicitly provided with the ambiguous nature of the input images, and therefore learns to extract this information to solve the task. In addition, the model learns to ignore top-down signals when the image is unambiguous and context is contradictory or irrelevant. Given that the apical compartment must combine contextual information and the contextually naive $\mu $ representation differentially based on input ambiguity, we hypothesized that solving the task effectively required a high gain modulation specific to the subset of context-relevant neurons. To assess this possibility, we computed the average amplitude of apical signals arriving at LRP subsets pertaining to predicting each of two input classes (A,B) and ambiguous AB for each scenario of matching, irrelevant or contradictory context (Fig. 4a to f). When comparing all input/context scenarios, we found that apical signals were highest when input images were ambiguous and context informative (1-ANOVA, F$_{3, 416}$=237.024, $P$ < 0.0001, for the main effect of input/context scenario; Fig. 4b). In the ambig.Match scenario, neurons most relevant for the target class were associated with the highest amplitude in the apical compartment (1-ANOVA, F$_{2, 417}$=17.9445, $P$ < 0.0001, for the main effect of neuron class; Fig. 4b). Apical input onto neurons outside of these defined subpopulations (other) remained low ($\sim $0.07 to 0.08) across all scenarios (data not shown), indicating there was no relationship between other neurons’ apical signal and the scenario.

We also computed the basal amplitude for the same subsets of neurons defined above and found that there was a higher average amplitude on the matching relative to the contradictory subpopulation for unambiguous stimuli (Fig. 4c). Likewise, there was a higher basal amplitude on the ambiguous subpopulation relative to other subpopulations for ambiguous inputs. Basal input onto the “other” subpopulation was found to be significantly lower for ambig.match and unambig.contra scenarios, but similar in magnitude to the unambig/ambig.irrel scenarios (<0.02, Fig. 4c).

These observations are indicative of a mechanism whereby ambiguous bottom-up representations are biased toward top-down representations by specific apical inputs Fig. 4d, whereas unambiguous bottom-up representations are preserved due to nonspecific top-down modulation Fig. 4e.

To evaluate if specific top-down modulation is necessary for solving the ambig.match task, we applied a mask on the apical inputs to the corresponding subpopulations post-training, and assessed the model’s ability to solve the task (Appendix 2, Fig. 2). Specifically, we compare test set accuracy when inputs are ambiguous and context is relevant, under the condition where context-relevant apical inputs are masked out (activations set to 0), compared with the condition where random apical inputs are masked. We also evaluate the effect of mask size and found that masking the apical inputs to context-relevant neurons specifically led to a steeper degradation of accuracy as we increase the number of masked inputs. This highlights the key role of these neurons’ apical compartments in integrating relevant contextual signals.

### Deriving context from temporal information

**Fig. 5:**
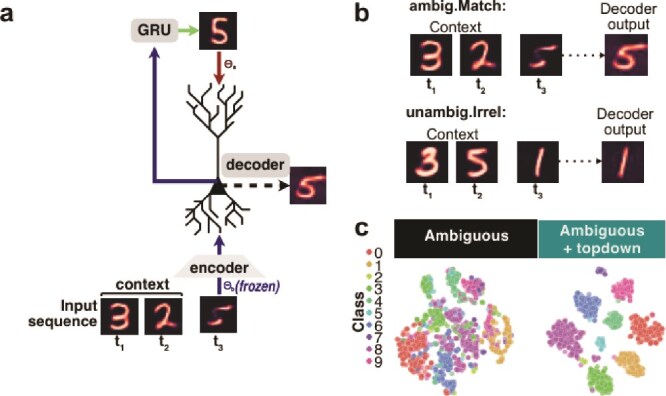
Deriving context from temporal information. (a), rationale for training a model that leverages context derived from temporal sequences through a recurrent network (GRU). (b), example sequence used to generate context signals (as the arithmetic sum of two digits, left and center columns) to resolve ambiguity of an input image (right column). (c), t-SNE projection of latent representation associated with ambiguous inputs before (left) and after (right) combining top-down signals.

Contextual priors can also be extracted from the temporal domain, specifically by leveraging past information to decode incoming ambiguous sensory inputs. To extend our model to cases where contextual signals are derived from the temporal domain, we trained a GRU network to predict the arithmetic sum of two unambiguous digits as encoded by our pretrained feedforward weights $\mathbf{\Theta _{b}}$. The final output state of the GRU was then given as a contextual signal for our contextual integration model (Fig. 5a; see Methods). We find that in cases where the input is ambiguous and the temporal sequence sums to a plausible interpretation of the ambiguous image, the model updates perceptual representations using contextual signals to successfully resolve ambiguities (Fig. 5b, top). Importantly, context is effectively ignored in cases where the input is unambiguous and the contextual signals are irrelevant (Fig. 5b, bottom). Using low-dimensional projections of latent representations, we find that top-down context derived from the temporal domain effectively disentangled overlapping representations when inputs are ambiguous, similar to the oracle case (Fig. 5c).

## Discussion

The ability to appropriately use and switch contexts to make sense of a vast stream of sensory information is an important feature of robust cognitive systems.

It is thought that in the neocortex, contextual representations in higher order regions modulate lower sensory regions through top-down interactions arriving on the apical dendrites of pyramidal neurons (Larkum 2013; Phillips 2017), an architectural bias we refer to here as the “apical prior.” While it is known that apical dendrites modulate neuronal firing, their specific computational role in cognitive processes such as contextual integration remains poorly understood.

Here, we tested whether the “apical prior” represents an architectural bias that is functionally useful for contextual integration in neural networks. To this end, we implemented (1) a neural network architecture with distinct apical compartments and a neuron-wise integration rule based on the observation that apical dendrites are gain modulators of somatic activity (Waters et al. 2003; Larkum et al. 2007), and developed (2) an ambiguous image classification task that requires contextual integration to be solved. We trained (1) the contextual modulator (top-down synapses onto apical dendrites) by gradient descent to simultaneously learn all input-context conditions defined in our task. In this setting, we found that this bio-inspired dendritic architecture outperformed a single-compartment model that processes both sensory and contextual information with a single nonlinear activation.

By applying LRP, we analyzed the amplitude of apical inputs onto class-specific neurons for ambiguous stimuli. We found the highest gain modulation in context-relevant neurons (matching the target class), consistent with the principles of biased competition (Spratling 2008).

Moreover, we found that the competition introduced by the contextual integration adapted to the level of ambiguity in the task: the top-down network learned to (a) refine representations of ambiguous stimuli by applying strong but sparse gain modulation to basal signals when contextual information is both available and relevant, and (b) provide weak and nonspecific modulation in the case of unambiguous stimuli and contradictory or irrelevant contexts, thus preserving the bottom-up representations.

### Comparison with existing computational frameworks

From a computational perspective, apical dendrites have been previously shown to support some types of contextual integration in biophysically realistic neurons and ANNs (George et al. 2020; Naumann et al. 2022; Wybo 2022; Baronig and Legenstein 2024). The tasks used in these previous works are multitask, single scenario problem settings, meaning that context (eg one-hot encoded task id) is always assumed to be relevant. Alternative approaches to flexible contextual integration have involved explicitly learning to prevent associations between irrelevant context and sensory inputs (Baronig and Legenstein 2024), which represents a successful example of how to handle spurious contexts in a simple task. As an extension of this approach, our model must infer how to handle the interaction between sensory input and context across distinct tasks and scenarios, where context is relevant, irrelevant, or contradictory, but without explicit knowledge of the task.

Crucially, we only found a significant advantage of the apical prior over single compartment integration in the scenario where inputs are unambiguous and context is contradictory (and should be ignored). This highlights a specific advantage for apical dendrites in the role of contextual integration, where accounting for varying contextual relevance is crucial to broadly robust performance. Furthermore, while we show that the task can be solved using either a gain modulation (Hadamard) or additive apical integration rule (Appendix 1 Table 4), we found that, under low contextual certainty, the Hadamard integration rule (apical prior) enabled the model to ignore contradictory contextual signals more effectively compared with additive apical integration.

Another key difference of our study with most previous work is that as basal and apical signals are combined post-activation, our rule can only amplify already active neurons, unlike Wybo (2022) and Naumann et al. (2022), but inline with experimental evidence (Larkum et al. 1999). Thus, our approach maintains the sparsity of the network representations, which can preserve selectivity, and would also be more metabolically efficient.

One notable prior work that applies context-dependent modulation to feedforward networks is context-dependent gating (XdG) (Masse et al. 2018), which implements contextual inputs by concatenation of context signals and gating task-specific neurons. The former could be comparable with our single compartment (basal-only) ablation as it employs a single zone of integration. The main difference with our basal-only ablation is that XdG (a) is not informed by intermediate sensory representations, and (b) as most prior works assumes task labels are given and that context always agrees with sensory input. Therefore, it is unknown if XdG, implemented as-is, can deal with cases where context should be overridden by sensory input, as in the unambig.Contra case.

In our experiments, the advantage of apical dendrites was revealed in our multiscenario contextual integration task. In order to compare our results with previous work, we examined the effect of our integration mechanism in a standard multitask setting (with task-ids provided to the network, 4). Under these conditions we found that all variants perform similarly well. This can be explained by the setting being simpler, whereas the distinct apical compartment shows its advantage under more varied and challenging types of contextual integration that require inference of ambiguity (no task ids provided).

While not explicitly modular, our framework could be incorporated into the Global Workspace Theory (see Goyal (2022) for a comparison of recent implementations), to provide a candidate mechanism for resolving inconsistency between modules under ambiguity. It is important to note that our architecture embodying the apical prior presupposes there is asymmetry for how contextual (top-down) and perceptual (bottom-up) representations are combined under ambiguity, which is included in some (eg Mittal (2020)) but not other (Goyal 2022) implementations of Global Workspace Theory. In general, modular representations can be learned in a distributed and scalable manner, with interactions between local populations (or modules). As highlighted in Goyal (2022), this frees up the capacity of a learning system for more tasks, as it can more efficiently identify task-relevant information and reuse what it has learned. The “apical prior” here implies that these interactions have an asymmetry: they can be contextual signals (in our case via top-down projections to apical compartments), or feed-forward/bottom-up signals. Here, we find that this asymmetry provides flexibility to solve perceptual tasks under diverse ambiguity and contextual scenarios.

The integration rule we employ—whether as a Hadamard product or summation—offers computational simplicity but oversimplifies the rich, nonlinear interactions observed in biological neurons. The interplay between basal and apical dendrites involves interactions between complex phenomena such as dendritic spikes and back-propagating action potentials, resulting in so-called back-propagation activated calcium spikes (BACs) that trigger a burst of spikes at the soma (Larkum et al. 1999). Notably, Larkum et al. (2004) showed that while these nonlinearities are highly pronounced under isolated conditions, they become smeared out when a neuron receives abundant synaptic input, as would occur in vivo. This results in input-output properties well approximated by apical gain modulation, as employed here. Alternative models of apical modulation, such as those incorporating gain and shift dynamics, have been explored in Tugsbayar et al. (2025). Wybo (2022) implemented gain and shift modulation in more biophysically realistic neurons, which included also NMDA spikes. They found that their network expressed a different apical dendritic state for each task, which in turn modulated rapid feedforward processing to solve a multitude of tasks. Future investigations should thus explore incorporating these elements to determine how a more biologically realistic nonlinearity might affect model performance under the ambiguous perception tasks proposed here.

### What is necessary for robust contextual integration?

Thus far, we have shown that in our contextual integration task, the apical prior provides a specific advantage compared with single-compartment integration when sensory or contextual signals are unreliable. Here, we extend these findings to make broader predictions for what specific components/computations might be necessary for robust contextual integration, and how such a mechanism could plausibly be implemented in the cortex.

Our results suggest that it is sub-optimal to sum all activity together in a single (basal) compartment, if sensory information and context can be contradictory, as would be the case for general cognitive systems. This highlights a key difference between single and two-zone integration of information. As such, an interesting experiment would be to compare the single vs two-zone integration of bottom-up representations and top-down contextual representations in in vivo experiments or simulations of neurons, which more faithfully respect the biophysical constraints of pyramidal neurons in the neocortex, to observe whether our results hold in a more biologically plausible setting, or if it is just a feature of ANNs and/or how they were trained here.

Furthermore, based on our results, we can infer that the apical compartment encodes some information about the ambiguity of the sensory input, and the relevance of context for a given sensory input. An interesting experiment could involve silencing or deactivating apical dendrites in more realistic neurons (biophysical simulations or in-vivo experiments), to assess the effect on accurate perception for our proposed contextual integration tasks.

Our analysis showed that mutual information between apical and basal activation is positive for the ambig.Match scenario. On the other hand, mutual information is close to 0 for the unambig.Irrel and unambig.Contra scenario (Appendix, Table 3). This is not surprising given we explicitly trained under the assumption that local representations, given matching unambiguous contextual information, should collapse to the target representation. Moreover, when sensory ambiguity is low, local representations should be invariant to contextual ambiguity, and mismatching contextual information. This is an important feature of the model supporting robust contextual integration given that most available contextual signals in rich, multimodal real-world sensory experiences are distractors for any one particular task, and should be discarded.

Following this, we predict that a model cannot learn the relevance of context without encountering and recognizing cases with irrelevant context during training (negative samples). Extending this idea to unsupervised learning, it is interesting to consider how the model could identify contextual relevance. In an unsupervised setting this could arise from an information-maximization learning rule like InfoNCE (Oord et al. 2019), which uses a contrastive loss with positive and negative pairs defined via augmentation of the input (positive, relevant) against other inputs (negative, irrelevant). Unfortunately, the creation of positive and negative pairs relies heavily on modality-specific knowledge to construct useful data augmentations (eg a rotation applied on an image). A more general approach will be necessary to create such contrastive pairs for many modalities in parallel.

An important feature of our model is that it could learn a mapping of top-down signals onto the apical compartment separately from the contextual representations. This is compatible with regions high in the cortical hierarchy, such as the hippocampus (via the entorhinal cortex), having independent mechanisms for learning (a) how they represent contexts, and (b) how those contextual representations should modulate the sensory representations of lower regions (Maren et al. 2013). Because we assume the stability of the sensory and contextual representation prior to training contextual integration, we cannot make any conclusions from the present study about how these two representations could be simultaneously co-learned. However, we speculate that in the neocortex, there should be some degree of stability in the sensory and contextual representations before learning to perform contextual integration.

This could be compatible with the distributed learning of modules (cortical regions) for sensory and contextual representations initially via self-supervised learning, prior to learning the contextual interactions between the modules (carried by cortico-cortical white matter projections).

### Limitations

While our model unifies the function of contextual integration with key biophysical properties of dendrites, some of our modeling assumptions and simplifications are worth discussing with respect to their empirical support. Firstly, unlike other computational frameworks such as George et al. (2020), which seek to incorporate the rich diversity and laminar architecture of the neocortex, our model is designed to implement some prominent features of cortical pyramidal neurons without focusing on a specific cortical layer, such as layer 2/3 or layer 5 pyramidal neurons. Specifically, we focus on the functional properties of two-zone dendritic integration. In the cortical circuit, these mechanisms appear to be conserved across pyramidal neurons in layers 2/3, 5, and 6 (Larkum et al. 1999; Waters et al. 2003; Larkum et al. 2007; Ledergerber and Larkum 2010). Here, we assume that contextual signals are provided by neurons in a higher region, which would likely correspond to outputs of layer 5 pyramidal neurons in that region, and integrated in the apical dendrites of putative layer 2/3, 4, or 5 pyramidal neurons (Bastos 2012; Schuman et al. 2021). It is worth noting that top-down projections may also innervate the basal compartment of layer 2/3 neurons, and the lower regions of layer 5 pyramidal neuron basal dendrites (Harris 2019), but this case was not considered in this study.

Furthermore, our study considers the importance of apical and basal zones, but does not consider oblique dendrites emanating from the apical shaft. These have also been shown in mouse visual cortex to have properties that are distinct from basal dendrites, such as linear vs supralinear integration and absence vs presence of NMDAR-mediated burst-timing synaptic potentiation, respectively (Yaeger 2024). Oblique dendrites are known to make up a considerable fraction of total dendritic length (Schaefer et al. 2003; Petanjek et al. 2008) and to modulate BAC firing that couples coincident apical and basal inputs (Schaefer et al. 2003). These findings imply oblique dendrites could function as a “third factor” in determining the impact of top-down projections on feed-forward sensory processing, and learning. However, the presence and role of such a third factor would be pyramidal-cell-type-specific. Anatomical studies in vibrissal cortex estimate that L3 PCs would receive feedforward thalamo-cortical input onto basals and top-down input onto apical tufts (Meyer 2010). In contrast, for pyramidal neurons in layer 5b, Yaeger (2024) found that their oblique dendrites receive thalamo-cortical inputs, whereas their basal dendrites receive no thalamo-cortical inputs. It is worth noting that Meyer (2010) inferred from axonal and dendritic overlap that L5b PCs in vibrissal cortex would receive input from higher order thalamus (generally top-down) and sensory thalamus on apical and proximal oblique dendrites, and basal and distal oblique dendrites, respectively. These findings differ from those of Yaeger (2024), and may reflect region-specific differences in innervation (vibrissal vs visual). It is an interesting future direction to consider the role oblique dendrites could play in contextual integration and learning within the cortical laminar architecture. This role likely differs across specific PC cell types (L3 vs L5), so future work would need to differentiate models for specific PC types.

It is also important to point out that our model assumes similar integration properties in basal and apical (summation); however dendritic topology and morphological features of its subtrees (Uylings and Pelt 2002) are known to influence integration (Rall 1994; Ooyen et al. 2002; Poirazi et al. 2003; Ujfalussy et al. 2018). Moreover, apical and basal zones can have different topologies, eg as has been shown in human prefrontal cortex (Petanjek et al. 2008).

Another missing feature in the current framework is local recurrence, which is well established in the neocortex and features prominently in biophysical models (Reimann 2024). The different PC types in the neocortical microcircuit innervate each other on different dendritic subdomains implementing intra- and inter-laminer recurrent excitation (Thomson and Bannister 2003; Narayanan 2015). Future work should investigate how the Hadamard product formalism developed here could be used to model such type-specific innervation domains and laminar interactions, and their functional relevance for sensory integration and learning. Moreover, governed by Dale’s principle, cortical recurrence due to pyramidal neurons and spiny interneurons is exclusively excitatory, whereas recurrence due to nonspiny interneurons (lacking apical dendrites) is exclusively inhibitory. This specialization of recurrent interactions may be important for implementing competitive coding, attractor dynamics, functional assemblies, and winner-take-all mechanisms, which are all relevant for computation with sparse representations. Although the optimal form of these dynamics for accurately modeling cortical function remains an active research area, our study focused on the contextual integration of individual pyramidal cells and the role of apical dendrites in ambiguous tasks. Our functional solutions trained using backpropagation, and assessed with LRP analysis revealed “functional assemblies” associated with input classes; however, these assemblies are purely input driven in the current model. Incorporating recurrent dynamics—including lateral inhibition—could enhance the emergence of such assemblies and offer a more general framework for processing contextual signals over time.

Despite these missing anatomical features, the framework developed here could serve to study the functional role of laminar architecture in the neocortex: Our study relies largely on the functional mechanism of two-zone integration in pyramidal neurons, which appears to be consistent across pyramidal neuron types, despite potential differences in the sources of the inputs they receive.

In this study we have used LRP to reveal functional assemblies and the differential role played by apical signals across ambiguous scenarios. Since the development of LRP (Binder et al. 2016), new explainability methods have been developed, such as Neuron Shapley (Ghorbani and Zou 2020), designed to capture interactions across neurons. Ideally, the functional assemblies identified by both approaches would significantly overlap, reinforcing their relevance to task performance. For future work, a hierarchical version of our model is under development, for which we could leverage these explainability approaches to identify sparse, functionally relevant subnetworks (assemblies) and assess the impact of apical dendrites at each level.

One potential criticism is that we train here using gradient descent, for which concerns have been raised as to its biological plausibility (Lillicrap et al. 2020). The aim of this study is not to find biological learning rules, but rather to understand contextual integration. We use gradient descent as a “best in class” general learning approach to find good solutions to our task, which allows us to analyze the learned representations and the respective contributions of apical and basal compartments.

Our framework lacks the implementation of biologically plausible plasticity rules. We leave to future work the exploration of solutions that could be obtained through such rules, such as Lee et al. (2015); Meulemans et al. (2022), and comparison with solutions we obtained via backprop. Indeed the analysis above suggests that information-maximization learning rules, along the lines of Hjelm (2019) but between basal and apical compartments, could be a promising direction.

### Outlook

As state-of-the-art deep learning models increase exponentially in size, many recent works focus on scalable, parameter-efficient fine-tuning for target tasks, a prominent example being low-rank adapters or LoRAs (Hu 2021; Chen et al. 2024). While LoRAs use resources efficiently, they lack a contextual integration component, and as such, they could be complementary to the “apical prior” architecture studied here. A future direction could combine LoRAs with the apical prior for efficient contextual integration tasks at scale.

Another important aspect of scaling state-of-the-art models is multimodality. Often in human brains, inputs from different modalities are processed simultaneously, and provide context to each other. As another promising future direction, we propose that our model could be extended to hierarchies of modules of sensory modalities interacting with each other. Deep learning models that implement multimodal interactions are an active area of research (Radford 2021; Alayrac 2022; Mustafa et al. 2022). While they excel at multimodal generation conditioned on one modality, they struggle with conflicting information or contradictions in mixed-modality data (Chen et al. 2024).

We speculate that the brain might employ both the apical prior and sparse weights to learn to efficiently handle the large pool of available sources of context across modalities. Toward an implementation of the Global Workspace Theory, we argue that (a) there must be a mechanism for resolving ambiguity or discrepancy between modules before the step in which modules compete to write to the shared global workspace (as in Goyal (2022)), and (b) implementation of the “apical prior” satisfies this requirement. Thus, this study provides an important step in understanding the process by which brain-wide networks of many modalities can coordinate and integrate information.

## Supplementary Material

CC_submission_supp_bhaf134

## Code availability

All experiments were carried out using PyTorch. We use the MNIST and EMNIST datasets, where number of output classes are $C = 10$ for MNIST, and $C = 26$ for EMNIST. The code for models and data analysis is publicly available under: https://github.com/ABL-Lab/expectation-clamp The code used to generate the ambiguous datasets as well as links to pregenerated datasets are available under: https://github.com/ABL-Lab/ambiguous-dataset.
