# Supplementary material for: Learning to combine top-down context and feed-forward representations under ambiguity with apical and basal dendrites
Source: Cereb Cortex. 2025 Jun 23;35(6):bhaf134. doi: 10.1093/cercor/bhaf134 (PMC12205961; doi:10.1093/cercor/bhaf134)
Supplement: CC_submission_supp_bhaf134 [file cc_submission_supp_bhaf134.pdf]

Cerebral Cortex, , pp. 1–4

doi: 10.48550/arXiv.2312.05484

LEARNING APICAL-BASAL CONTEXTUAL INTEGRATION

Learning to combine top-down context and feed-forward representations under ambiguity with apical and basal dendrites

Nizar Islah,[1,2,5] Guillaume Etter,[1,5] Mashbayar Tugsbayar,[4,5,6] Busra Tugce Gurbuz,[5,7] Blake Richards[3,4,5,6,8] and Eilif B. Muller[1,2,5]

[1]Centre de Recherche Azrieli du CHU Ste-Justine, Université de Montréal, 3175 Chem. de la Côte-Sainte-Catherine, H3T 1C5, Montréal, Canada, [2]Département d'informatique et de recherche opérationnelle, Université de Montréal, 3150 Jean Brillant St, H3T 1N8, Montréal, Canada, [3]School of Computer Science, McGill University, 3480 Rue University, H3A 2A7, Montréal, Canada, [4]Department of Neurology and Neurosurgery, McGill University, 1033 Pine Avenue West, H3A 1A1, Montréal, Canada, [5]Mila Quebec AI Institute, 6666 Rue Saint-Urbain, H2S 3H1, Montréal, Canada, [6]Montreal Neurological Institute, McGill University, 3801 Rue University, H3A 2B4, Montréal, Canada, [7]Department of Quantitative Life Sciences, McGill University, 550 Sherbrooke W., H3A 1E3, Montréal, Canada and [8]CIFAR Learning in Machines and Brains Program, 661 University Ave., Suite 505, M5G 1M1, Toronto, Canada

[*]Corresponding author 1: EBM. eilif.muller@umontreal.ca [*]Corresponding author 2: NI. nizar.isl17@gmail.com

FOR PUBLISHER ONLY Received on 24 November 2024; revised on 21 March 2025; accepted on 20 April 2025

Abstract

© The Author . Published by Oxford University Press. All rights reserved. For permissions, please e-mail: journals.permissions@oup.com

Supplementary Material

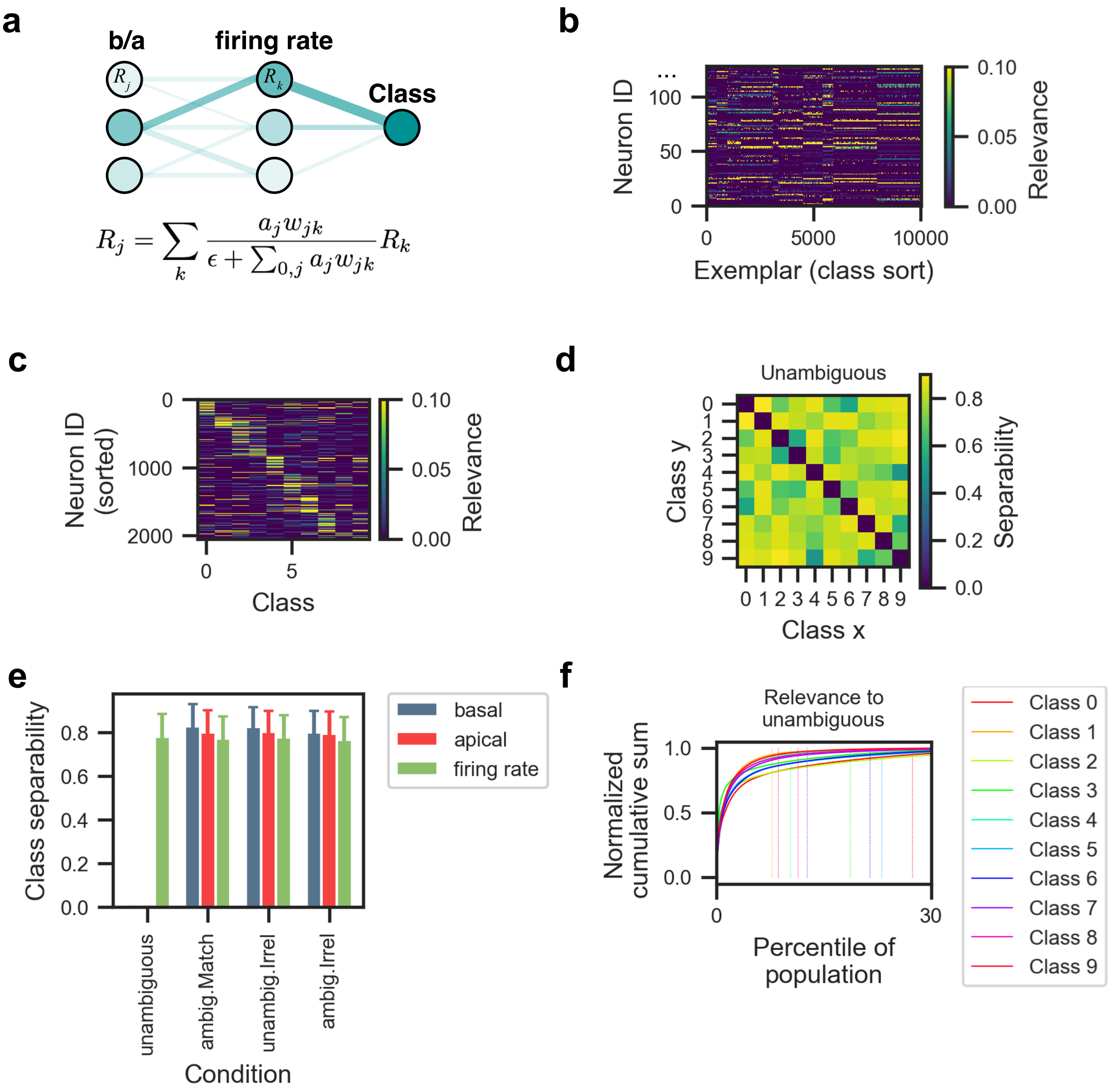

Fig. 1. Rationale for identification of single neuron groups. **a**, we computed relevance of neurons and their compartments (basal, apical, firing rate) using LRP. **b**, Relevance of each neuron (soma) for input images sorted by class label. **c**, average relevance for each neurons (ascending sort) for each class. **d**, representational separability for every pair of classes. **e**, class separability for each training scenario, and per dendritic compartment (blue, basal; red, apical; green, firing rate). **f**, normalized cumulative sum of relevance for ranked neurons and for each class. Vertical dashed lines indicate the minimum number of neurons required to preserve 95% of all relevance for each class.

modulation	context_only	backbone	scenario_labels	ambig.Match	unambig.Match	unambig.Irrel	ambig.Irrel	unambig.Contra
Hadamard	no	ae	no	94.9 ± 0.8	98.4 ± 0.2	95.7 ± 0.1	46.9 ± 1.2	95.0 ± 0.1
Hadamard	no	vae	no	94.2 ± 1.4	98.9 ± 0.2	96.2 ± 0.4	47.1 ± 0.8	94.9 ± 0.5
	no	vae	yes	94.7 ± 0.6	99.2 ± 0.2	96.7 ± 0.2	47.2 ± 0.8	97.9 ± 0.2
	yes	vae	no	67.2 ± 0.8	97.3 ± 0.8	95.0 ± 0.5	47.6 ± 0.2	91.8 ± 1.7
	yes	vae	yes	76.4 ± 1.8	99.2 ± 0.1	96.5 ± 0.4	47.7 ± 0.4	97.9 ± 0.3
sum	no	vae	no	99.1 ± 0.0	98.9 ± 0.3	96.2 ± 0.2	47.2 ± 0.6	94.3 ± 0.6
	no	vae	yes	99.7 ± 0.1	99.3 ± 0.2	96.4 ± 0.2	47.2 ± 1.3	97.8 ± 0.2
	yes	vae	no	79.3 ± 1.3	97.9 ± 0.4	95.3 ± 0.4	47.3 ± 0.6	92.3 ± 1.3
	yes	vae	yes	86.8 ± 0.5	99.1 ± 0.2	96.6 ± 0.1	47.6 ± 0.4	97.9 ± 0.2
concat	no	vae	no	97.7 ± 0.8	99.0 ± 0.1	96.4 ± 0.2	47.1 ± 0.3	95.1 ± 0.6
	no	vae	yes	98.9 ± 0.7	99.3 ± 0.1	96.6 ± 0.1	47.6 ± 0.5	98.0 ± 0.0
	yes	vae	no	78.6 ± 1.6	98.1 ± 0.4	95.6 ± 0.1	47.1 ± 0.7	93.2 ± 0.1
	yes	vae	yes	84.9 ± 1.2	99.2 ± 0.1	96.5 ± 0.1	46.9 ± 0.3	97.9 ± 0.1

Table 1. EMNIST test set accuracy across various metrics with different context representations, modulation strategies, and scenario labels. Results are expressed for each scenario as mean % ± standard deviation with 3 random seeds.

modulation	context_only	backbone	scenario_labels	ambig.Match	unambig.Match	unambig.Irrel	ambig.Irrel	unambig.Contra
Hadamard	no	ae	no	98.4 ± 0.7	99.1 ± 0.1	98.3 ± 0.2	47.8 ± 0.2	97.3 ± 0.2
Hadamard	no	vae	no	98.5 ± 0.3	98.5 ± 0.1	97.9 ± 0.1	46.0 ± 0.8	96.0 ± 0.4
	no	vae	yes	99.0 ± 0.4	99.7 ± 0.0	99.1 ± 0.1	46.3 ± 0.9	99.4 ± 0.2
	yes	vae	no	86.8 ± 1.0	94.0 ± 0.5	92.6 ± 0.7	46.8 ± 0.5	85.7 ± 0.9
	yes	vae	yes	91.7 ± 1.2	99.6 ± 0.1	99.1 ± 0.1	45.4 ± 0.7	99.4 ± 0.2
sum	no	vae	no	99.6 ± 0.1	98.1 ± 0.3	97.4 ± 0.3	46.2 ± 0.6	94.7 ± 0.6
	no	vae	yes	99.9 ± 0.0	99.5 ± 0.1	99.0 ± 0.0	46.7 ± 0.1	99.2 ± 0.1
	yes	vae	no	82.6 ± 4.0	96.2 ± 1.7	95.7 ± 1.6	47.1 ± 0.8	93.4 ± 2.3
	yes	vae	yes	89.6 ± 2.5	99.6 ± 0.2	99.1 ± 0.2	46.2 ± 0.2	99.4 ± 0.2
concat	no	vae	no	98.8 ± 0.5	98.1 ± 0.2	97.4 ± 0.4	46.0 ± 0.4	94.1 ± 0.6
	no	vae	yes	99.2 ± 1.1	99.6 ± 0.1	99.1 ± 0.2	47.1 ± 0.4	99.4 ± 0.2
	yes	vae	no	81.1 ± 1.2	96.7 ± 1.0	95.9 ± 1.3	46.7 ± 0.3	93.8 ± 2.2
	yes	vae	yes	87.5 ± 0.8	99.7 ± 0.1	99.1 ± 0.1	46.7 ± 0.4	99.4 ± 0.1

Table 2. MNIST test set accuracy across various metrics with different context representations, modulation strategies, and scenario labels. Results are expressed for each scenario as mean % ± standard deviation with 3 random seeds.

		**Mutual Information***	
**Scenario**	**Modulation**	**Mean**	**Std**
**ambig.Match**	**Hadamard**	0.114	0.017
	**sum**	0.107	0.020
**unambig.Contra**	**Hadamard**	-0.004	0.004
	**sum**	-0.004	0.005

Table 3. Mutual Information metrics for ambig.Match and unambig.Contra with Hadamard and sum operations. *We report MI as an approximate measure. This is computed by the expected information gain (which is equivalent to MI) on the readout predictions with and without top-down modulation given context. Since our readout prediction is a non-linear mapping from the apical and basal compartments, this is an approximate measure, as MI is only invariant to linear transformations.

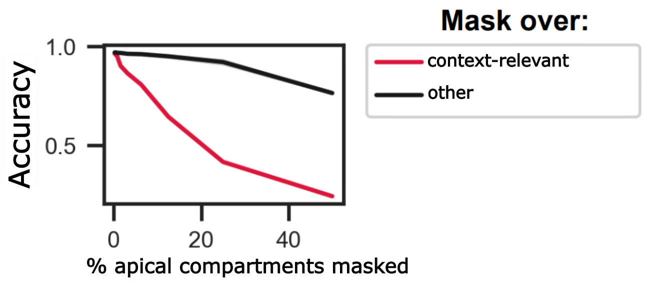

Fig. 2. Masking analysis. We compare test set accuracy when inputs are ambiguous and context is relevant (ambig.Match) with context-relevant apical inputs are masked out (activations set to 0), compared to the condition where random apical inputs are masked. Masking the context-relevant apical inputs specifically led to a steeper degradation of accuracy as the number of masked inputs increases.
